# Tale of an Unsound Horse

**Published:** 1896-08

**Authors:** 


### CORRESPONDENCE.

---

### TALE OF AN UNSOUND HORSE.

THE big brown trotting-horse, "Sheraldine," a bold looker and a fair actor when speeded, was not long since sold at auction in this horse-market (Chicago) for $1650 as a heavy harness-horse, Mr. John Dupee being the buyer. Gen. Joseph T. Torrence, also of this city, was the runner up. The horse was sold as sound, and Mr. Dupee availed himself of the buyer's privilege of having his purchase examined for soundness before accepting him. Dr. Joseph Hughes, who was for a long time the *Gazette's* veterinarian, inspected the gelding for Mr. Dupee, and threw him back on the seller, whereupon there was a great row. Four distinguished "vets" in this city passed the horse as sound, and it is said that Gen. Torrence signified his willingness to accept him at his last bid—$1600. But "Sheraldine" is not in Gen. Torrence's barn, neither does he keep company with Mr. Dupee's high-steppers; he remains in the sale-stable from which he was first offered. Dr. Hughes, canny Scot that he is and careful practitioner, found a well-marked case of scirrhous (indurated) cord, the result of recent bungling castration, and a clearly marked fistulous condition was present, with flow of pus. Such a condition is remedied only by a delicate and dangerous operation. Of course it is not observable on casual examination; indeed, it requires a search to find it, but nothing escapes the eye of Dr. Hughes.

So vigorous was the criticism of the little Scotchman by the dealers and their "vets" that Mr. Dupee's ire was roused. He secured the names of a number of America's most eminent veterinarians and obtained their opinions as to whether such a condition as existed in "Sheraldine" should be considered disqualifying unsoundness in a sale-ring. Armed with their affirmative replies indorsing Dr. Hughes's opinion in every particular, Mr. Dupee placed $5000 in his inside pocket and journeyed to the yards to "take a fall" of about that size out of the dealer. But the wary salesman declined the wager. That Mr. Dupee wanted the horse and wanted him badly his bid of $1650 proves; and that his veterinarian advised him intelligently and accurately as to the soundness of the horse he believes has been thoroughly demonstrated. And this is the tale of an unsound horse.—*Breeders' Gazette*, May 13, 1896.

